# Mass-Spectrometry-Based Approaches in Natural Products Research: Progress, Challenges, and Future Perspectives

**DOI:** 10.3390/plants15101422

**Published:** 2026-05-07

**Authors:** Felipe Moura A. da Silva, Rita de Cássia S. Nunomura

**Affiliations:** 1 Coordenação de Tecnologia e Inovação, Instituto Nacional de Pesquisas da Amazônia (INPA), Manaus 69067-375, AM, Brazil; 2 Departamento de Química, Universidade Federal do Amazonas, Manaus 69080-900, AM, Brazil

Natural products research has undergone a profound transformation over the past decades, driven not only by technological advances but also by a shift in the conceptual frameworks that guide the field. Traditionally rooted in reductionist approaches that emphasize the isolation of individual compounds and linear structure–activity relationships, natural products research is increasingly moving toward integrative and systems-level perspectives. This transition reflects a broader epistemological shift, in which natural products are no longer viewed as isolated chemical entities, but as components of complex biological and ecological networks that mediate interactions across multiple levels of organization [1]. Within this evolving context, mass-spectrometry (MS)-based approaches have emerged as central tools for exploring chemical diversity. Techniques such as liquid chromatography–mass spectrometry (LC–MS) and gas chromatography–mass spectrometry (GC–MS) now enable the rapid detection and relative quantification of thousands of metabolites in a single experiment. As a result, MS-based metabolomics has become a cornerstone of contemporary natural products research, allowing for the high-throughput analysis of complex biological matrices and facilitating the discovery of bioactive compounds. However, these advances have also introduced new analytical and conceptual challenges, particularly in the context of data processing, metabolite annotation, and biological interpretation.

One of the defining features of modern MS-based metabolomics is the generation of large and complex datasets. A single LC–MS experiment can produce gigabytes of data, containing thousands of spectral features that must be processed, aligned, and interpreted [2,3]. The scale of these datasets has made manual analysis impractical, leading to the development of computational workflows that integrate feature extraction, statistical analysis, and metabolite annotation. These workflows have significantly expanded the analytical capacity of the field, enabling researchers to move from targeted analyses toward global, untargeted exploration of metabolomes. At the same time, the reliance on automated data processing and spectral matching has raised important questions regarding the reliability and reproducibility of metabolite identification.

In this context, metabolite annotation remains one of the most critical and challenging steps in MS-based studies. While advances in spectral databases, molecular networking, and in silico prediction tools have improved annotation capabilities, a substantial proportion of detected features still correspond to unknown or only putatively identified compounds. Importantly, the assignment of chemical identities based solely on spectral similarity or computational inference does not necessarily provide definitive structural confirmation. Recognizing these limitations, community-driven efforts such as the Metabolomics Standards Initiative have established guidelines for reporting metabolomics data, emphasizing the importance of metadata, quality control, and confidence levels in metabolite identification [4]. These standards play a crucial role in promoting transparency, reproducibility, and comparability across studies.

Despite the increasing sophistication of MS-based approaches, it is essential to acknowledge that these techniques do not replace classical strategies in natural products chemistry. Rather, they complement them. In addition to their well-established role in metabolite annotation and dereplication, MS-based approaches are now widely applied to investigate dynamic metabolic processes, correlate chemical profiles with biological activity, support quality control, and guide extraction and optimization strategies. Nevertheless, while metabolomics and related workflows are highly effective for prioritizing samples, identifying known compounds, and generating biologically relevant hypotheses, definitive structural elucidation often still requires compound isolation followed by spectroscopic characterization using techniquessuch as nuclear magnetic resonance (NMR). This is particularly important in cases involving novel structures and ambiguous annotations, or when precise structure–activity relationships are needed. Therefore, a balanced approach that integrates high-throughput MS-based screening with targeted isolation and characterization remains fundamental for robust natural products research.

Within this context, the thirteen papers published in this Special Issue illustrate how mass-spectrometry-based approaches are being applied in practice, while also reflecting the current capabilities and limitations of these strategies across a diverse range of applications. Several studies employ metabolomic approaches to correlate chemical profiles with biological activity, with multivariate statistical analyses applied in some cases to support data interpretation. For example, the work by Sales et al. explored seasonal variations in essential oil composition from the aerial parts of *Vernonanthura polyanthes* (Asteraceae) and their correlation with antileishmanial activity, identifying key metabolites associated with bioactivity through multivariate modeling [5]. On the other hand, studies on plant extracts and natural matrices demonstrated how MS-based annotation combined with biological assays could reveal compounds with relevant bioactivities. For example, hydroalcoholic extracts of *Fridericia platyphylla* leaves were profiled by LC–MS/MS, enabling the annotation of phenolic compounds, including flavonoid glycosides, flavone aglycones, and phenolic acid derivatives, which were associated with antiproliferative and anti-migratory effects against cervical cancer cell lines [6]. Moreover, avocado honey was characterized in terms of phenolic composition and antioxidant potential using LC–MS/MS, revealing a high phenolic content and strong radical-scavenging activity, along with the annotation of bioactive compounds such as caffeine, scopoletin, and abscisic acid [7]. Broader analyses of honey samples from different botanical origins in the Italian market further identified a wide diversity of phenolic compounds and volatile markers, with quantitative variations associated with the botanical source and a positive correlation between phenolic content and antioxidant activity [8]. In addition, honey produced by tbe Melipona species from the Amazonas State in the Brazilian Amazon were analyzed using GC–MS, enabling the identification of characteristic volatile profiles associated with regional floral sources, and were evaluated for antioxidant and moderate antimicrobial activity against selected microbial strains [9].

Beyond chemical–biological correlations, other contributions demonstrate the application of MS-based workflows to investigate broader metabolic relationships and dynamic biological processes. For instance, UHPLC–MS/MS combined with molecular networking was used to explore the metabolic overlap between the endophytic *Penicillium* species and their host plant, *Annona jahnii*, revealing shared and analogous metabolites and providing insights into biosynthetic capabilities [10]. In parallel, metabolomic approaches have been applied to investigate dynamic biochemical processes, such as cultivar-dependent metabolic changes during fruit ripening. For example, the transition from C7 sugars to C6 sugars was monitored across different ripening stages in avocado, revealing distinct metabolic patterns among cultivars and highlighting the capacity of MS-based techniques to capture metabolic variations across biological conditions [11].

In a complementary approach, Otsuki et al. used high-resolution Orbitrap MS to profile daphnane diterpenoids in the flower buds and blooming flowers of *Daphne odora*, identifying a diverse set of compounds, including previously unreported structures. Among these, daphneodorin I was isolated and its structure was elucidated through extensive physicochemical and spectroscopic analyses, representing the first daphnane diterpenoid with a *Z*-configured phenolic acyl moiety reported from plants of the Thymelaeaceae family [12]. Extending this strategy of MS-guided isolation, the integration of MS-based metabolomics with bioactivity-guided fractionation was demonstrated in the study of *Piper longum* fruit, where metabolomic profiling guided the identification and isolation of bioactive lignans and amide alkaloids, including previously unreported compounds with SIRT1-modulating activity [13]. At an earlier stage of this workflow, the optimization of extraction methods combined with MS-based profiling and molecular networking has also been shown to play a critical role in improving the recovery and characterization of bioactive compounds from plant matrices, as demonstrated in the study of *Licaria armeniaca* [14].

In addition, several studies demonstrate the application of MS-based techniques in quality control, food chemistry, and authentication. Braga et al. applied direct-injection MS combined with chemometric analyses to investigate the botanical origin of “miraruira”, a medicinal plant-based product widely commercialized in street markets in the Amazonas State (Brazil), addressing uncertainties related to its authenticity and highlighting implications for product standardization and therapeutic consistency [15]. Furthermore, quantitative approaches combining LC–HRMS and GC-based methods were employed to assess the lipid composition and variability in macauba (*Acrocomia aculeata*) oils, enabling the detailed characterization of fatty acid profiles and triacylglycerol species across commercial samples. The study revealed significant compositional variability and deviations from expected profiles, highlighting the relevance of MS-based techniques for the quality control, authentication, and industrial applications of plant-derived oils [16]. Finally, review-based contributions provide a broader perspective on chemical diversity and MS-based profiling, as illustrated by the comprehensive analysis of uncommon flavonoid subclasses from the Fabaceae family, integrating mass spectrometric characterization with pharmacological evaluation and in silico predictions of ADMET properties, thereby highlighting their largely unexplored bioactive potential [17].

Together, these contributions illustrate the versatility of MS-based methodologies, and their expanding role across multiple domains of natural products research, from chemical ecology and metabolomics to pharmacological evaluation, food science, and methodological development. At the same time, they also highlight persistent challenges that continue to shape the field. Among these, the accurate annotation of metabolites, the integration of chemical data with biological function, and the standardization of analytical workflows remain central issues. The increasing reliance on large-scale datasets further emphasizes the need for robust data curation, rigorous validation strategies, and transparent reporting practices. Without such measures, there is a risk of propagating uncertain or incorrect annotations, ultimately compromising downstream biological interpretations.

Looking forward, future developments in natural products research will likely be shaped by the continued integration of analytical chemistry, bioinformatics, and systems biology. Advances in artificial intelligence and machine learning are expected to enhance spectral interpretation and annotation processes, while expanding and improving spectral databases will increase the reliability of compound identification. However, the effectiveness of these tools will depend critically on the quality of the underlying data and the adoption of standardized methodologies. In parallel, there is a growing need to bridge the gap between chemical ecology and pharmacognosy, moving toward a more holistic understanding of natural products as mediators of biological interactions rather than merely sources of bioactive compounds [1]. In this context, future research should prioritize integrative strategies that combine high-throughput analytical techniques with rigorous validation approaches. This includes not only improving annotation confidence through orthogonal methods but also incorporating ecological, biochemical, and functional data to better contextualize chemical diversity. Ultimately, the challenge is not only to generate more data but to generate more meaningful and reliable knowledge from these data.

In conclusion, mass-spectrometry-based approaches have revolutionized natural products research, enabling unprecedented access to chemical diversity and accelerating the pace of discovery. However, these advances must be accompanied by critical reflection and methodological rigor. By acknowledging both the strengths and limitations of MS-based strategies, and by fostering integrative and standardized approaches, the field can move toward a more robust, reproducible, and comprehensive understanding of natural products in their biological and ecological contexts.

## Data Availability

Not applicable.
